# Combination of a 15-SNP Polygenic Risk Score and Classical Risk Factors for the Prediction of Breast Cancer Risk in Cypriot Women

**DOI:** 10.3390/cancers13184568

**Published:** 2021-09-11

**Authors:** Kristia Yiangou, Kyriacos Kyriacou, Eleni Kakouri, Yiola Marcou, Mihalis I. Panayiotidis, Maria A. Loizidou, Andreas Hadjisavvas, Kyriaki Michailidou

**Affiliations:** 1 Department of Cancer Genetics, Therapeutics and Ultrastructural Pathology, The Cyprus Institute of Neurology and Genetics, Nicosia 2371, Cyprus; kristiay@cing.ac.cy (K.Y.); kyriacos@cing.ac.cy (K.K.); mihalisp@cing.ac.cy (M.I.P.); loizidou@cing.ac.cy (M.A.L.); 2 The Cyprus School of Molecular Medicine, The Cyprus Institute of Neurology and Genetics, Nicosia 2371, Cyprus; 3 Department of Medical Oncology, Bank of Cyprus Oncology Center, Nicosia 2012, Cyprus; eleni.kakouri@bococ.org.cy (E.K.); yiola.marcou@bococ.org.cy (Y.M.); 4 Biostatistics Unit, The Cyprus Institute of Neurology and Genetics, Nicosia 2371, Cyprus

**Keywords:** breast cancer, polygenic risk score, classical risk factors, risk prediction, Cypriot women

## Abstract

**Simple Summary:**

Breast cancer is the most commonly diagnosed type of cancer in women worldwide. Stratification of women based on their individual breast cancer risk could guide targeted preventative strategies and population screening. Integrated models that combine the effects of a polygenic risk score (PRS) with classical breast cancer risk factors could provide an individualized breast-cancer risk estimation. Although various studies have extensively evaluated the performance of such integrated models in populations of European ancestry, no previous studies have included individuals of Greek-Cypriot origin. To this end, we have assessed the predictive performance of a 15-SNP PRS (PRS_15_), in combination with classical breast-cancer risk factors, in women of Greek-Cypriot origin. This proof-of-concept study suggests that models combining genetic data with classical risk factors may be used in the future for the prediction of breast-cancer risk and, therefore, supports their potential clinical utility for targeted preventative strategies in Cypriot women.

**Abstract:**

The PRS combines multiplicatively the effects of common low-risk single nucleotide polymorphisms (SNPs) and has the potential to be used for the estimation of an individual’s risk for a trait or disease. PRS has been successfully implemented for the prediction of breast cancer risk. The combination of PRS with classical breast cancer risk factors provides a more comprehensive risk estimation and could, thus, improve risk stratification and personalized preventative strategies. In this study, we assessed the predictive performance of the combined effect of PRS_15_ with classical breast-cancer risk factors in Cypriot women using 1109 cases and 1177 controls from the MASTOS study. The PRS_15_ was significantly associated with an increased breast cancer risk in Cypriot women OR (95% CI) 1.66 (1.25–2.19). The integrated risk model obtained an AUC (95% CI) 0.70 (0.67–0.72) and had the ability to stratify women according to their disease status at the extreme deciles. These results provide evidence that the combination of PRS with classical risk factors may be used in the future for the stratification of Cypriot women based on their disease risk, and support its potential clinical utility for targeted preventative actions and population screening.

## 1. Introduction

Breast cancer is the most commonly diagnosed type of cancer in women around the world [1]. Disease susceptibility varies between individuals, and is influenced by multiple genetic and non-genetic risk factors such as age, height, BMI, reproductive and menstrual history, use of hormone replacement therapy and lifestyle risk factors [2,3,4,5,6], all of which confer individually a moderate effect on breast cancer risk. Family history is one of the most established risk factors associated with breast cancer predisposition. So far, multiple breast cancer susceptibility variants have been discovered. These include pathogenic variants in high-risk and moderate-risk genes, which are rare in the population, and account for about 20–25% of the excess familial relative risk (FRR) of breast cancer. Large-scale genome-wide association studies (GWAS) identified more than 200 SNPs common in the population, each individually conferring a small effect on the disease risk, but collectively account for about ~18.3% of the excess FRR of the disease [7,8,9,10].

Individualized risk estimation could be used for the stratification of women into different categories according to their breast cancer risk, which could potentially guide targeted risk management strategies, and improve population screening efficiency [11,12]. A polygenic risk score (PRS) combines multiplicatively the effects of common susceptibility variants and could be used for the stratification of women according to their personal breast cancer risk [13,14,15,16,17]. Recently, Mavaddat et al. 2019 have constructed a PRS including 313 SNPs for the prediction of overall, ER-positive and ER-negative, breast cancer risk in women of European ancestry [14]. Compared with females in the 40–60% quintile of the PRS_313_ risk distribution, females in the 1st and 99th percentiles had 0.27-times and 4-times increased overall breast cancer risk, respectively [14]. Combination of PRS with classical risk factors can substantially improve the prediction of breast cancer risk and could detect individuals at different levels of the disease risk [18,19,20,21,22,23,24]. Furthermore, the incorporation of PRS into breast cancer risk prediction models such as BOADICEA, Tyrer–Cuzick, Gail and Rosner–Colditz can improve their discrimination power [25,26,27,28,29], and provide a more comprehensive individualized risk estimation [30].

Although large studies have assessed the performance of such combined models in populations of European descent, no previous study included individuals of Greek-Cypriot origin. In Cyprus, an island in the Mediterranean region, more than 500 new breast cancer cases are diagnosed in females annually, accounting for around 35% of all female cancer cases [31]. Currently, little is known about how common variants influence breast cancer susceptibility in Cypriot women or about the utility of their combined effect (PRS) for the prediction of breast cancer risk. A previous study, by our group, has evaluated 11 SNPs identified through the initial GWAS for association with breast cancer risk in Cypriot women and concluded that four of them were nominally significantly associated with breast cancer risk [32]. Furthermore, a previous study evaluated the strength of associations between classical risk factors and breast cancer risk in Cypriot women [33].

The aim of this study was to combine a PRS_15_ with classical breast cancer risk factors and assess its predictive power in Cypriot women using samples from the MASTOS study [33]. 

## 2. Materials and Methods

### 2.1. Study Population 

Study participants included 2286 females derived from the MASTOS study [33]. MASTOS is a population-based case-control study that includes 1109 female breast cancer cases with mean age (SD) at interview 55.99 (9.15), and 1177 female healthy controls with mean age (SD) 55.65 (7.04) of self-reported Greek-Cypriot origin. Cases were females who were diagnosed with breast cancer between January 1999 and December 2006. Healthy controls were participating in the National program for breast cancer screening, with negative mammography results, and no previous personal history of breast cancer. Demographic and phenotypic data of all the participants were collected using a specially designed questionnaire, through a standardized interview. All study participants were recruited from January 2004 to December 2006. Detailed information on the purpose, design of the study, data collection and study population is described elsewhere [33]. Three samples were excluded from the analysis due to the high missing rate of phenotypic data. Therefore, the total number of individuals included in the analysis was 2283, consisting of 1174 controls and 1109 cases.

The Cyprus National Bioethics Committee approved the study protocol (EEBK/ΕΠ/2005/08), and all study subjects gave written consent. The study was conducted in compliance with the Helsinki Declaration. 

### 2.2. SNP Selection and Genotyping 

Fifteen SNPs that were previously identified via GWAS (prior to 2013) [34,35,36,37,38,39,40] were selected and genotyped in all MASTOS study participants, using the Taqman SNP genotyping assays from Applied Biosystems Inc. (ABI), according to the manufacturer’s instructions and as described in detail elsewhere [32]. Detailed information about the 15 SNPs included in this study is summarized in Table 1.

### 2.3. Statistical Analysis 

Allele frequencies, odds ratios (ORs) and 95% confidence intervals (CIs) of the 15 SNPs were calculated in the MASTOS study using logistic regression analysis in R (version 3.6.3) [41]. A 15-SNP PRS model (PRS_15_) was subsequently constructed, and a score was created for each woman using the following Equation (1), as previously described in Pharoah et al. 2002 [42]:

PRS = β_1_x_1_ + … + β_k_x_k_ + β_15_x_15_ (1)

Briefly, in this Equation (1), β_k_ is the log OR of the minor allele for SNP_k_ obtained from the iCOGS study [7,8], and x_k_ is the number of minor allele copies that are carried by each individual for SNP_k_ and can take values 0, 1 or 2 (minor allele was defined based on the published minor allele frequency (MAF)). Information about the published ORs and allele frequencies of the 15 SNPs included in the PRS is summarized in Table 1. 

Logistic regression analysis was performed to evaluate for associations between PRS_15_ and breast cancer risk, and by quartiles of the PRS_15_ risk distribution, standardized by the controls, and using the 2nd quartile, 25–50%, as the reference. All calculations were carried out in R (version 3.6.3).

Associations between each risk factor and breast cancer risk were calculated using univariable logistic regression analysis (Table S1). Pair-wise Spearman correlations were calculated in the control group to assess for interactions between the PRS_15_ and 10 classical breast cancer risk factors including: menopausal status (yes/no), age at menarche (years), parity (yes/no), age at first full-term pregnancy (FFTP) (per 5 years), breastfeeding among parous women (yes/no), height (cm), BMI (kg/m^2^), use of hormone-replacement therapy (HRT) (yes/no), smoking status (yes/no) and family history (in a first degree relative) (yes/no).

Subsequently, a multivariable model consisting of the PRS_15_ and the risk factors was constructed. Multivariable logistic regression analysis was performed to determine the association between the integrated risk model and breast cancer risk. Then, MASTOS dataset was divided into deciles according to the predicted risk probability of the integrated risk model, to evaluate its ability to stratify women based on their disease status. Logistic regression analysis was performed to generate OR (95% CI) of each decile, by using the 5th decile as the reference. For the final integrated risk model, only individuals with complete observations were used for the analysis. Thus, the total number of individuals included in the final analysis was 1780, consisting of 900 controls and 880 cases. Sensitivity and specificity analysis were performed to evaluate the performance of the model.

The global goodness-of-fit of each model was evaluated using the Hosmer–Lemeshow test. The area under the receiver operating characteristic curve (AUC) and 95% DeLong CI were calculated in order to evaluate the discrimination power of the models, using the pROC package in R [43]. All tests were two-sided, using a *p*-value threshold of 0.05, and were carried out in R (version 3.6.3).

## 3. Results

### 3.1. Evaluation of the PRS_15_ and Its Association with Breast Cancer Risk in Greek-Cypriot Women 

Single SNP analysis showed that 4 of the 15 SNPs were associated with breast cancer risk at a nominal significant *p*-value < 0.05 (Table 1), and in total 11 of the 15 SNPs had the point estimates of the ORs in the same direction, as previously reported in the iCOGS study (indicated in bold in Table 1) [7,8]. Subsequently, a PRS consisting of these 15 SNPs was constructed.

**Figure 1:**
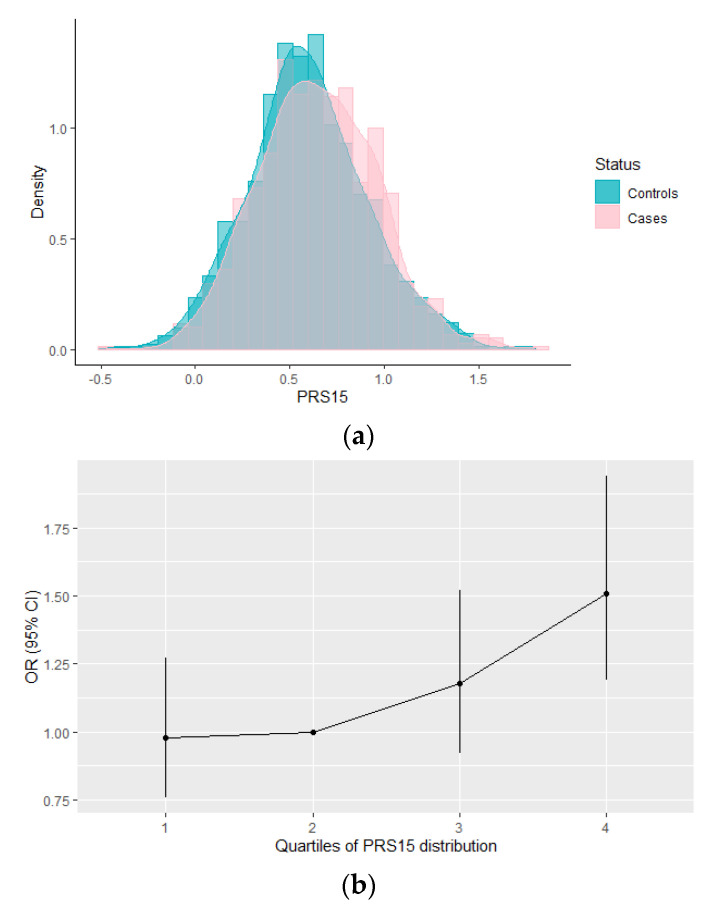
Distribution of the PRS_15_ and its association, by quartiles, with breast cancer risk in the MASTOS study. (**a**) Distribution of the PRS_15_ in controls (blue) and in cases (pink) in Cypriot women. The average PRS_15_ was higher in cases compared with the controls (**b**) OR (95% CI) for breast cancer risk by quartiles of PRS_15_ risk distribution, using as reference the 2nd quartile (25–50%). Women in the fourth quartile had a significant increased breast cancer risk compared with those in the 2nd quartile.

PRS_15_ distribution plots are shown for both controls (blue) and cases (pink) in Figure 1. The average PRS_15_ was higher in cases [mean (SD) = 0.645 (0.312)] compared to controls [mean (SD) = 0.595 (0.316)] (Figure 1a). The PRS_15_ was, statistically, significantly associated with increased breast cancer risk in Cypriot women with OR (95% CI) 1.66 (1.25–2.19) and *p*-value = 0.0004. The AUC (95% CI) of PRS_15_ was 0.55 (0.52–0.57) and was well-calibrated (Hosmer–Lemeshow test x^2^ = 11.77, *p*-value = 0.162). As illustrated in Figure 1b, compared with females in the 2nd quartile (25–50%) of the PRS_15_ risk distribution, the estimated OR (95% CI) for those in the first quartile was 0.98 (0.76–1.27), *p*-value = 0.88, whereas for those in the fourth quartile was 1.51 (1.19–1.94), *p*-value = 0.0009 (Figure 1b; Table S2A). The estimated OR of PRS_15_ did not change substantially when adjusted by age or family history (Table S2B).

### 3.2. Association between the Integrated Risk Model Consisting of PRS_15_ and Classical Risk Factors with Breast Cancer Risk in Greek-Cypriot Women

**Figure 2:**
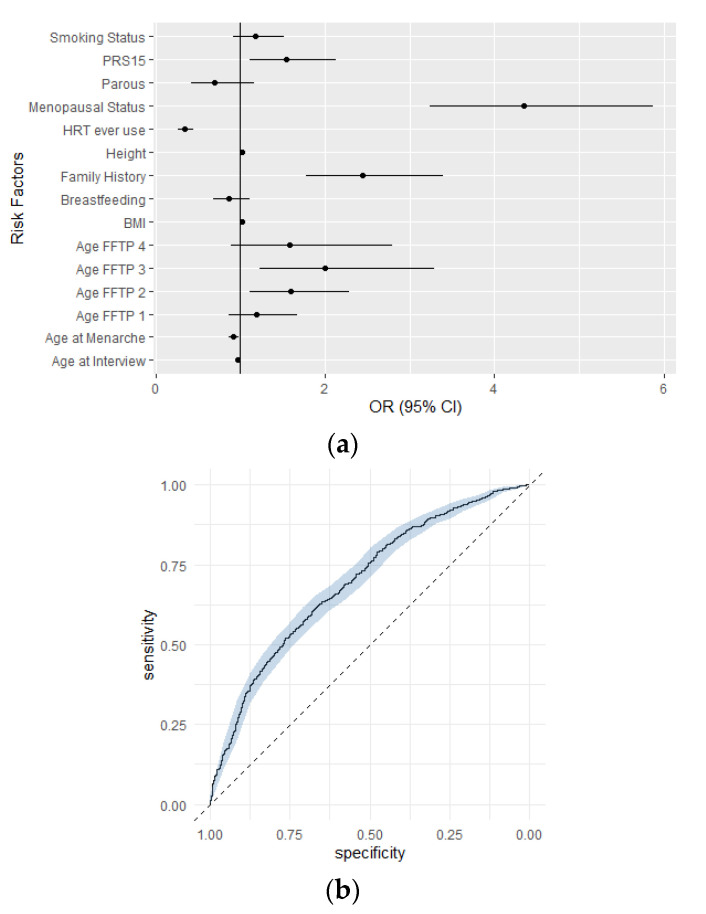
Association of the integrated risk model with breast cancer risk in the MASTOS study: (**a**) Associations between risk factors included in the final integrated risk model with breast cancer risk in the MASTOS study. Estimated ORs (95% CI) of each risk factor for breast cancer risk are illustrated; Age FFTP category 5 (Nulliparous) was included in the category 0 of Age FFTP (reference). (**b**) ROC curve for the integrated risk model, (AUC 0.70, 95% CI 0.67–0.72).

We further proceeded with the evaluation of a multivariable model including breast cancer risk factors, that were previously used in larger studies, and in combination with PRS_15_. Table S1 summarizes the distribution of classical risk factors in the MASTOS study. There was no evidence of interactions between PRS_15_ and any of the classical breast cancer risk factors (Table S3). Thus, an integrated risk model that included all risk factors and PRS_15_ was constructed. The integrated risk model achieved a risk discrimination score of AUC (95% CI) 0.70 (0.67–0.72) and was well calibrated (Hosmer-Lemeshow x^2^ = 8.73, *p*-value = 0.37) (Figure 2, Table S4). Sensitivity of the model was 0.644 and specificity was 0.624.

**Figure 3:**
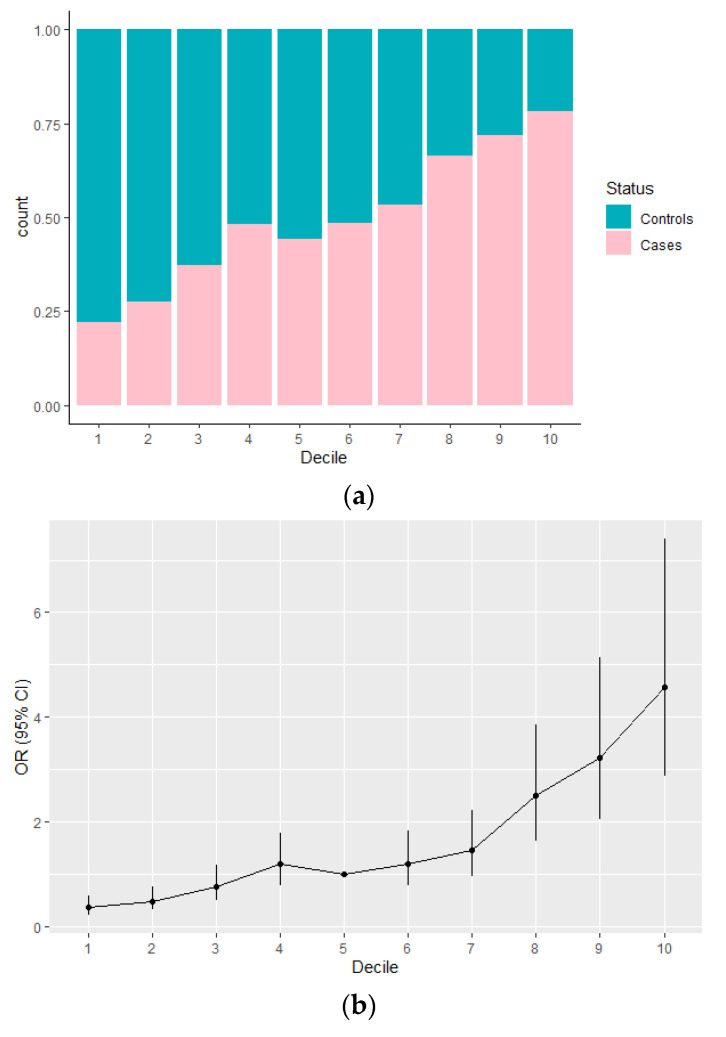
Association between the integrated risk model and breast cancer risk in the MASTOS study (**a**) Distribution of controls (blue) and cases (pink) of the MASTOS study in each decile, when the dataset was divided based on the predicted risk probability of the integrated risk model; (**b**) ORs (95% CI) by decile for breast cancer risk, using the 5th decile as the reference.

To assess the ability of the integrated risk model to discriminate individuals based on their disease status, MASTOS dataset was divided into deciles based on the predicted risk probability of the integrated risk model. As illustrated in Table 2, compared with the 5th decile, the estimated OR (95% CI) in the first decile was 0.36 (0.22–0.57), with *p*-value = 1.55 × 10^−5^, and included 15.4% of controls, and 4.4% of cases. Similarly, the estimated OR (95% CI) in the 2nd quartile was 0.48 (0.31–0.75), with *p*-value = 0.001 and included 15% of controls and 5.8% of cases. In contrast, the estimated OR (95% CI) in the 9th decile was 3.22 (2.04–5.13) with *p*-value = 6.46 × 10^−7^ and included 5% of controls and 13% of cases, and in the last decile the estimated OR (95% CI) was 4.58 (2.88–7.4), with *p*-value = 2.44 × 10^−10^ and included 4.2% of controls and 15.6% of cases (Table 2, Figure 3). 

## 4. Discussion

Prevention and early detection of breast cancer are key objectives in the clinical management of the disease. The incorporation of PRS into risk prediction models that include classical breast cancer risk factors can potentially provide a more comprehensive personalized breast cancer risk estimation and, thus, has potential clinical applications in guiding targeted population screening and personalized preventative strategies. Although large studies have evaluated the performance of PRS and risk prediction models in populations of European ancestry, specific assessment among smaller European populations has not been extensively performed. Recent studies highlight the need of country-specific calibration of such risk prediction models for a more precise population-specific personalized risk estimation and classification [19,27,44].

The main aim of this study was to assess the predictive performance of a PRS consisting of 15 previously identified breast cancer susceptibility variants in combination with other classical breast cancer risk factors, and to evaluate its ability to discriminate Greek-Cypriot women based on their breast cancer risk. Hence, we demonstrated that the PRS_15_ was associated with an increased breast cancer risk in Greek-Cypriot women, and the integrated risk model had the ability to stratify Greek-Cypriot women based on their disease status at the extreme deciles. The results of this validation study support the potential clinical utility of a combined risk estimation model that will include PRS and classical risk factors for providing individualized breast cancer risk information and guiding targeted screening and preventative actions in our population.

To date, more than 200 common, low-risk SNPs have been discovered through GWAS to be associated with breast cancer predisposition in women of European ancestry, and a PRS_313_ has been constructed for the prediction of breast cancer risk in European women [9,14]. In our study, we included 15 SNPs identified by GWAS, before 2013, which have higher effect sizes compared to most of the recently discovered SNPs and constructed a PRS. Of the 15 SNPs included in the PRS only a small number (4/15) were individually associated with breast cancer risk at nominal significant *p*-value in our study population, with the effect size of a larger number of the SNPs (11/15) being in the same direction, as previously described in the iCOGS study [7,8]. Based on this observation, the discriminatory ability of the combined effect of these variants was investigated. PRS_15_ was significantly associated with an increased breast cancer risk in Greek-Cypriot women, with OR (95% CI) of 1.66 (1.25–2.19), which falls within the range of ORs of the published PRSs constructed and evaluated in women of European descent, according to the Polygenic Score (PGS) Catalog [45]. The AUC (95% CI) of PRS_15_ was 0.55 (0.52–0.57), which was slightly lower compared with previous studies in European populations where the AUC ranged between 0.58–0.65 [30]. Incorporation of additional susceptibility SNPs in the PRS could potentially improve its discrimination power. Despite, the modest predictive accuracy of PRS_15_, women in the highest quartile of the PRS_15_ risk distribution had a statistically significant 1.5-times greater breast cancer risk compared to the average. 

Similar to other studies, we did not identify any significant interactions between the PRS and the other classical breast cancer risk factors (Table S3, Figure S1) [20,24,46]; thus, a multivariable model consisting of the PRS_15_ and all the risk factors was constructed. Combination of PRS_15_ with classical risk factors achieved a discrimination score of AUC (95% CI) 0.70 (0.67–0.72), and had the ability to stratify women based on their disease status at the extreme deciles which are the most important for risk-stratified preventative strategies (first decile included 15.4% of controls, and 4.4% of cases, whereas the last decile included 4.2% of controls and 15.6% of cases). Our results, provide evidence that such integrated risk models have the capacity to stratify Greek-Cypriot women based on their personal breast cancer risk. 

These results are consistent with previously published studies, demonstrating that integrated risk models, including a PRS, and classical breast cancer risk factors have the ability to stratify women according to their disease risk [19,20,24,26]. Recently, Triviño et al. 2020 have evaluated the predictive performance of an integrated risk model consisting of a PRS_92_ and 5 phenotypic risk factors in a cohort of Spanish women. Similarly to our results, the integrated risk model had the ability to stratify women according to their disease risk at the extreme deciles, and had a slightly higher predictive power compared to our study, AUC (95% CI) 0.80 (0.77–0.83) [22]. Additionally, van Veen et al. (2018) have used a PRS incorporating the effects of 18 SNPs, 9 of which were identical to the SNPs included in our analysis while another 5 were surrogate variants within the same gene/locus. The study concluded that the incorporation of PRS_18_ within the Tyrer–Cuzick model in combination with mammographic density, could substantially improve risk prediction accuracy, with AUC (95% CI) 0.67 (0.62–0.71) [26]. 

The main limitation of our study is the small number of SNPs included in the PRS. The inclusion of the PRS into a model with only including the classical risk factors did not make a substantial increase in the predictive performance of the model. In the future, larger studies, and incorporation of additional SNPs in the PRS, as well as additional risk factors in the integrated risk model, such as mammographic density, are needed for defining the best tool to be used in our population. 

In this study, we evaluated the predictive performance of the combined effect of a PRS with classical risk factors for the prediction of breast cancer risk in Greek-Cypriot women. Despite the limited number of SNPs included in the risk prediction model, our study highlights that it is worth assessing further the clinical utility of PRS for Greek-Cypriot women. 

## 5. Conclusions

In this study, we evaluated the predictive performance of a polygenic risk score consisting of 15 previously identified SNPs, in combination with classical breast cancer risk factors in women of Greek-Cypriot origin. Although the PRS and risk prediction models are extensively evaluated in individuals of European ancestry, no previous study included women from our population. This study demonstrates that polygenic information in combination with classical risk factors has the power to stratify Greek-Cypriot women based on their disease risk. These results suggest the potential clinical utility for the use of a combined model for the prediction of breast cancer risk in Cyprus.

## Figures and Tables

**Table 1:** Information about the 15 SNPs selected and genotyped in all MASTOS study participants.

CHR ^1^	SNP		Position ^2^	Alleles ^3^	MAF ^4^	iCOGS OR ^5^	iCOGS *p*-Value ^6^	MASTOS MAF ^7^	MASTOS OR ^8^	MASTOS *p*-Value
1	rs11249433		121280613	A/G	0.4	1.09 (1.07–1.12)	$4.43\times 10^{-20}$	0.46	1.00 (0.89–1.12)	0.98
**2**	**rs13387042**		**217905832**	**A/G**	**0.49**	**0.88 (0.86–0.9)**	** $8.91\times 10^{-41}$ **	**0.45**	**0.85 (0.75–0.95)**	**0.005**
3	rs4973768		27416013	C/T	0.47	1.1 (1.08–1.12)	$4.65\times 10^{-22}$	0.45	0.89 (0.78–1.00)	0.055
**5**	**rs889312**		**56031884**	**A/C**	**0.28**	**1.12 (1.1–1.15)**	** $2.87\times 10^{-27}$ **	**0.29**	**1.18 (1.04–1.34)**	**0.01**
**6**	**rs2046210**		**151948366**	**G/A**	**0.34**	**1.08 (1.06–1.1)**	** $2.13\times 10^{-14}$ **	**0.41**	**1.13 (1.00–1.27)**	**0.047**
**8**	**rs13281615**		**128355618**	**A/G**	**0.4**	**1.1 (1.08–1.12)**	** $3.26\times 10^{-22}$ **	**0.48**	**1.07 (0.95–1.20)**	**0.26**
**9**	**rs1011970**		**22062134**	**G/T**	**0.17**	**1.06 (1.03–1.08)**	** $2.68\times 10^{-5}$ **	**0.19**	**1.15 (0.99–1.33)**	**0.07**
**10**	**rs2981582**		**123352317**	**G/A**	**0.38**	**1.26 (1.24–1.28)**	** $1.6\times 10^{-120}$ **	**0.44**	**1.16 (1.03–1.31)**	**0.01**
**10**	**rs10995190**		**64278682**	**G/A**	**0.16**	**0.86 (0.83–0.88)**	** $1.6\times 10^{-29}$ **	**0.14**	**0.97 (0.82–1.15)**	**0.7**
**10**	**rs704010**		**80841148**	**C/T**	**0.38**	**1.08 (1.06–1.1)**	** $2.94\times 10^{-15}$ **	**0.37**	**1.01 (0.90–1.14)**	**0.83**
11	rs3817198		1909006	T/C	0.31	1.07 (1.05–1.09)	$1.09\times 10^{-10}$	0.31	0.97 (0.85–1.09)	0.59
**11**	**rs614367**		**69328764**	**C/T**	**0.15**	**1.21 (1.18–1.24)**	** $1.5\times 10^{-45}$ **	**0.11**	**1.09 (0.91–1.31)**	**0.36**
**16**	**rs3803662**		**52586341**	**G/A**	**0.26**	**1.24 (1.21–1.26)**	** $2.71\times 10^{-86}$ **	**0.33**	**1.01 (0.89–1.14)**	**0.86**
**17**	**rs6504950**		**53056471**	**G/A**	**0.28**	**0.94 (0.92–0.96)**	** $8.15\times 10^{-9}$ **	**0.26**	**0.94 (0.82–1.07)**	**0.34**
21	rs2823093		16520832	G/A	0.27	0.93 (0.91–0.95)	$2.39\times 10^{-12}$	0.73	1.07 (0.94–1.23)	0.28

^1^ Chromosome. ^2^ Build 37 position. ^3^ Reference/Effect allele. ^4^ Mean frequency of the effect allele in the controls taken from the iCOGS study [7,8]. ^5^ Per allele odds ratio (95% Confidence Intervals) for the effect allele taken from the iCOGS study (Associations for overall breast cancer). ^6^ *p*-value taken from the iCOGS study. ^7^ Frequency of the effect allele in controls in the MASTOS study. ^8^ Per allele odds ratio (95% Confidence Intervals) for the effect allele in the MASTOS study. Eleven SNPs in the same direction as previously reported in the iCOGS study are shown in bold.

**Table 2:** Total number of controls and cases of the MASTOS study included in each decile, when the dataset was divided based on the predicted risk probability of the integrated risk model. The estimated ORs (95% CI) of each decile for breast cancer risk were generated from logistic regression using the 5th decile as the reference.

Decile.	Controls (%)	Cases (%)	OR (95% CI)	*p*-Value
1	139 (15.4)	39 (4.4)	0.36 (0.22–0.57)	1.55 × 10^−5^
2	135 (15)	51 (5.8)	0.48 (0.31–0.75)	0.001
3	108 (12)	64 (7.3)	0.75 (0.49–1.16)	0.2
4	98 (10.9)	91 (10.3)	1.18 (0.78–1.79)	0.44
5	94 (10.4)	74 (8.4)	1	-
6	92 (10.2)	86 (9.8)	1.19 (0.78–1.82)	0.43
7	89 (9.9)	102 (11.6)	1.46 (0.96–2.21)	0.08
8	62 (6.9)	122 (13.9)	2.5 (1.63–3.86)	3.17 × 10^−5^
9	45 (5)	114 (13)	3.22 (2.04–5.13)	6.46 × 10^−7^
10	38 (4.2)	137 (15.6)	4.58 (2.88–7.4)	2.44 × 10^−10^

## Data Availability

The data presented in this study are available on request from ahsavvas@cing.ac.cy. The data are not publicly available due to ethical and privacy restrictions.

## Supplementary Materials

The following are available online at https://www.mdpi.com/article/10.3390/cancers13184568/s1, Figure S1: Heatmap of Spearman Pairwise Correlation between all the risk variables included in the analysis, in the control group of the MASTOS study population, Table S1: Phenotypic characteristics in the MASTOS study, Table S2A: Odds ratio (95% CI) by quartiles of PRS_15_ risk distribution generated using the 2nd quartile (25–50%) as the reference, Table S2B: PRS_15_ adjusted by family history and age, Table S3A: Table of Spearman Pairwise Correlation r between risk variables in the MASTOS study, Table S3B: Table of Spearman Pairwise Correlation *p*-value between risk variables in the MASTOS study, Table S4: Associations between the risk factors of the final integrated risk model with breast cancer risk in Cypriot women.
