# Supplementary material for: Combination of a 15-SNP Polygenic Risk Score and Classical Risk Factors for the Prediction of Breast Cancer Risk in Cypriot Women
Source: Cancers (Basel). 2021 Sep 11;13(18):4568. doi: 10.3390/cancers13184568 (PMC8468424; doi:10.3390/cancers13184568)
Supplement: Supplementary file 1 [file cancers-13-04568-s001.zip › cancers-1346105-Figure S1.pdf]

cancers

MDPI

Supplementary Materials: Combination of a 15-SNP Polygenic Risk Score and Classical Risk Factors for the Prediction of Breast Cancer Risk in Cypriot Women

Kristia Yiangou, Kyriacos Kyriacou, Eleni Kakouri, Yiola Marcou, Mihalis I. Panayiotidis, Maria A. Loizidou, Andreas Hadjisavvas and Kyriaki Michailidou

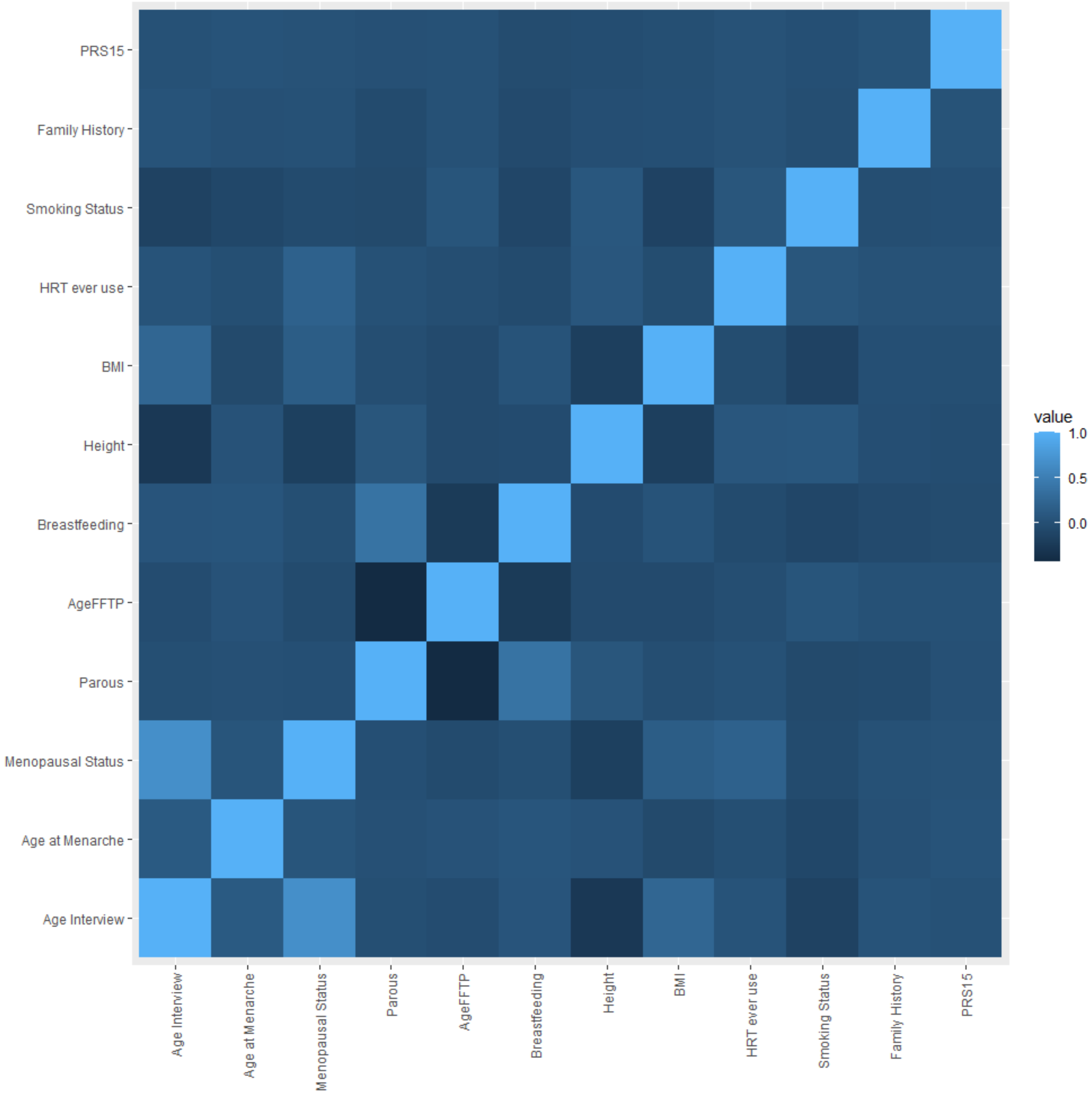

Figure S1. Heatmap of Spearman Pairwise Correlation between all the risk variables included in the analysis, in the control group of the MASTOS study population.
